# A Review of the Inflammatory Chorioretinopathies: The White Dot Syndromes

**DOI:** 10.1155/2013/783190

**Published:** 2013-10-31

**Authors:** Courtney M. Crawford, Okezie Igboeli

**Affiliations:** ^1^Department of Ophthalmology, Blanchfield Army Community Hospital, 650 Joel Drive, Fort Campbell, KY 42223, USA; ^2^Department of Ophthalmology, Madigan Army Medical Center, 9040 Fitzsimmons Drive, Joint Base Lewis-McChord, Tacoma, WA 98431, USA

## Abstract

The white dot syndromes are a group of inflammatory chorioretinopathies of unknown etiology which have in common a unique and characteristic appearance of multiple yellow-white lesions affecting multiple layers of the retina, retinal pigment epithelium (RPE), choriocapillaris, and the choroid. They also have overlapping clinical features. We discuss acute retinal pigment epitheliopathy, multiple evanescent white dot syndrome, acute posterior multifocal placoid pigment epitheliopathy, multifocal choroiditis and panuveitis, acute zonal occult outer retinopathy, birdshot chorioretinopathy, and serpiginous choroidopathy. Some of these diseases are associated with a viral prodrome suggesting a possible viral/infectious etiology, while others are associated with a number of systemic processes suggesting an autoimmune etiology. We also review the presentation, evaluation/diagnosis, and treatment of these entities as well as the prognosis. Where applicable we discuss recent advancements in diagnosing and treating the white dot syndromes.

## 1. Introduction

White dot syndromes is a term that has come into use over time to describe a group of inflammatory chorioretinopathies, not necessarily related to each other in pathogenesis, management, or prognosis. The common feature is a superficial resemblance of the lesions to each other. Nevertheless, the term conjures in the minds of readers a group of diseases and their unique features. Inflammatory chorioretinopathies, referred to as “white dot syndromes,” are of unknown etiology and typically affect young, healthy adults. Common presenting symptoms include photopsias, blurred vision, floaters, and visual field loss [1]. Many syndromes are characterized by a viral prodrome. The presence of discrete white lesions are located at various levels of the retina, outer retina, RPE, choriocapillaris, and choroid depending on the class of white dot condition [1]. While the etiology of white dot syndromes remains unknown, some suggest an autoimmune/inflammatory cause triggered by an exogenous agent. Some debate that the white dot syndromes are a spectrum of the same disease process, while others debate that each is a separate disease entity [2]. Despite the white dot similarities, most can be distinguished by the natural history, lesion morphology and progression, and fluorescein angiography pattern. 

## 2. Acute Retinal Pigment Epitheliitis (ARPE, Krill Disease)

Acute retinal pigment epitheliitis (ARPE) is a unilateral condition that occurs in otherwise healthy young adults in the second to fourth decade. Typically, this benign and self-limited condition resolve in 6–12 weeks with excellent visual acuity [3]. Most patients complain of mild visual loss and central metamorphopsia. On examination, small hyperpigmented lesions in a yellow halo configuration are present in the macula and paramacular area. The fundus findings in this first week are not reported; however, 1-2 weeks after the onset of symptoms are the lesions observed [3]. Fluorescein angiography shows early hyperfluorescence in a halo pattern and late staining. Visual field testing may show a central scotoma. Electroretinography is normal, and EOG may be abnormal, indicating that the disease processes is located at the level of the retinal pigment epithelium [1]. Choroidal neovascularization does not develop. Following recovery of central vision loss, the retinal pigment epithelium lesions are often not noticeable. No treatment is required for most cases of ARPE.

## 3. Multiple Evanescent White Dot Syndrome (MEWDS)

Multiple evanescent white dot syndrome (MEWDS) is a unilateral condition that typically affects healthy women of age 20–50 years old. One-half of patients typically have a viral prodrome, and most are moderate myopes. While MEWDS is mostly a unilateral process, bilateral cases of MEWDS have been described. Patients present with blurred vision, shimmering photopsias, dyschromatopsia, and a paracentral and often temporal scotomas [4]. 

On examination, vision may vary from 20/20 to 20/400. A relative afferent pupillary defect may be present. While the anterior segment is often void of signs of inflammation, mild vitreous cells are often present, and the optic nerve may be hyperemic. Characteristic of MEWDS, the posterior pole has multiple, discrete white spots at the level of the retina pigment epithelium or outer retina. The fovea has a pathognomonic granular appearance that may persist even after the inflammation has subsided [4]. 

Fluorescein angiogram displays punctuate hyperfluorescent lesions in a wreathlike configuration. The optic disc may show hyperemia in the late phase of the fluorescein angiogram. ICG angiography also shows round hypofluorescent spots in the posterior and midperipheral fundus; however, these spots may be more numerous than in the fluorescein angiogram [4]. Visual field testing displays a characteristic enlarged blind spot; however, temporal and paracentral scotomas may also be present with testing [4]. Electroretinogram may show a reduction of a-wave amplitude. Like the ERG, the EOG may also be abnormal; however, both studies typically normalize as the disease resolves [5]. 

Based on fluorescein angiography and electrophysiologic studies, it is shown that the most affected layers in MEWDS are the retinal pigment epithelium and the photoreceptors decreased a-wave [5]. Based on ICG angiography, the hypofluorescent lesions suggest that the choroidal circulation is also affected. Other associations with MEWDS include acute macular neuroretinopathy, acute zonal occult outer retinopathy, and acute idiopathic blind spot enlargement syndrome [4]. 

Vision is restored in 7–10 weeks, and recurrences are uncommon (see Table 1). Most cases resolve spontaneously without treatment, and vision is restored to a patient's baseline [4]. Although rare, recurrent cases of MEWDS have been treated effectively with cyclosporine, with no recurrence while on therapy [6]. Additionally infrequent incidence of CNV has been treated with success by intravitreal ranibizumab [7]. Visual field loss to include enlargement of the blind spot and visual phenomena of photopsias and dyschromatopsia may persist [4]. 

## 4. Acute Posterior Multifocal Placoid Pigment Epitheliopathy (APMPPE) 

Acute posterior multifocal placoid pigment epitheliopathy (APMPPE) is a bilateral condition that presents in otherwise healthy adults aged 20–30 years. In one-third of the patients, this condition occurs in conjunction with an influenza-like illness that may include meningeal symptoms [6]. Typically, men and women are equally affected. Patients usually present with asymmetric visual loss associated with a central and paracentral scotoma. Often times, the fellow eye is affected only days later; sometimes the onset may be delayed by several weeks [6]. 

**Figure 1:**
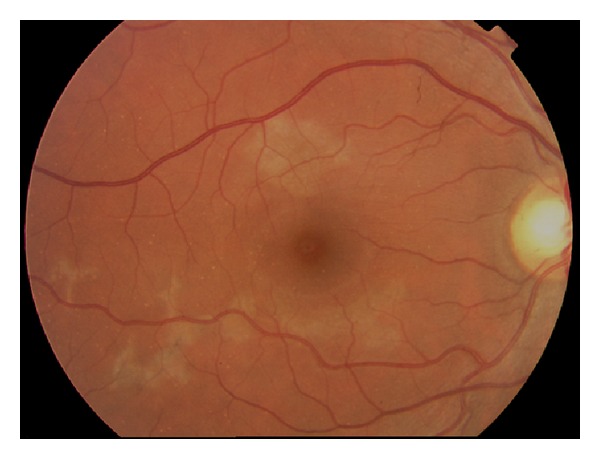
Photo courtesy of the University of California, San Francisco, Department of Ophthalmology.

On examination, the anterior chamber is quiet with few vitreous cells. Funduscopic evaluation reveals multiple, flat, cream-colored, placoid lesions at the level of the outer retina, retinal pigment epithelium, and choriocapillaris [6].  Figure 1 shows the fundus photo of a patient with APMPPE. The placoid lesions are usually less than one disc diameter in size and are mostly limited to the posterior pole. In 1-2 weeks time, these acute lesions may fade and are gradually replaced by various degrees of retinal pigment epithelium atrophy and hyperpigmentation [6]. 

**Figure 2:**
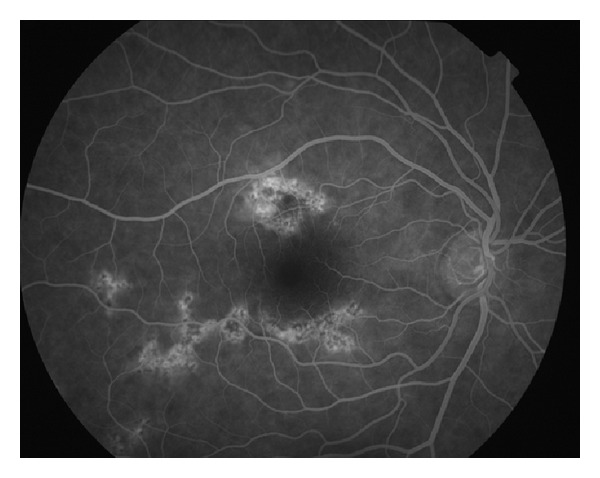
Photo courtesy of the University of California, San Francisco, Department of Ophthalmology.

 Diagnosis is based upon a characteristic “block early, stain late” fluorescein angiogram pattern.  Figure 2 shows late staining in a patient with APMPPE. Some sources state that the early hypofluorescence is related to both choroidal nonperfusion and the subretinal pigment epithelial lesions. Other sources deny choroidal nonperfusion, noting that the findings with ICG are not different than with fluorescein [7]. An abnormal electrooculogram may also be seen on electrophysiologic testing [6]. 

The etiology of APMPPE is presumed secondary to an abnormal immune response to an inciting agent. The medical literature provides strong evidence that APMPPE is caused by a delayed type hypersensitivity (DTH) reaction [8]. DTH is a type IV hypersensitivity that is caused by activation of sensitized T lymphocytes. This sensitization of T lymphocytes is explained by the occurrence of APMPPE following influenza vaccination, varicella vaccination, and antihepatitis B vaccine [9, 10]. In addition to vaccinations, APMPPE has been associated with several conditions to include mumps, sarcoidosis, Wegener's granulomatosis, polyarteritis nodosa, Lyme disease, ulcerative colitis, tuberculosis, and HLA subtypes B7 and DR2. Recurrent disease is theorized to be triggered by a hypersensitivity to antimicrobial agents [9]. 

No treatment is generally recommended because visual recovery of 20/40 or better is achieved in most cases. Factors that may contribute to a poorer prognosis include foveal involvement, older age, unilateral disease, and recurrent disease [6]. Rare cases of recurrent APMPPE resemble serpiginous choroiditis and have been called “ampiginous choroiditis” or relentless placoid retinochoroiditis [11]. If foveal involvement or central nervous system vasculitis is present, systemic corticosteroid treatment is recommended. Due to risk of CNS vasculitis, a systemic review of systems is important [12]. Symptoms of severe headache or meningeal symptoms warrant neuroimaging and further neurologic workup. Fatalities due to cerebral vasculitis have been associated with APMPPE [12]. 

## 5. Multifocal Choroiditis and Panuveitis (MCP)

Multifocal choroiditis is a bilateral condition that predominately affects women between ages 20 and 60 years old. Although classically a panuveitis, it is categorized among other white dot syndromes due to its characteristic funduscopic appearance. Patients typically present with decreased vision, an enlarged blind spot, and photopsias [13]. The etiology of multifocal choroiditis is unknown; however, some propose that antigens become sensitized in the retinal photoreceptors and retinal pigment epithelium by an exogenous pathogen. Uncertainty also remains regarding the classification of multifocal choroiditis, punctate inner choroidopathy, and diffuse subretinal fibrosis as separate diseases or a spectrum of the same disease [14]. 

**Figure 3:**
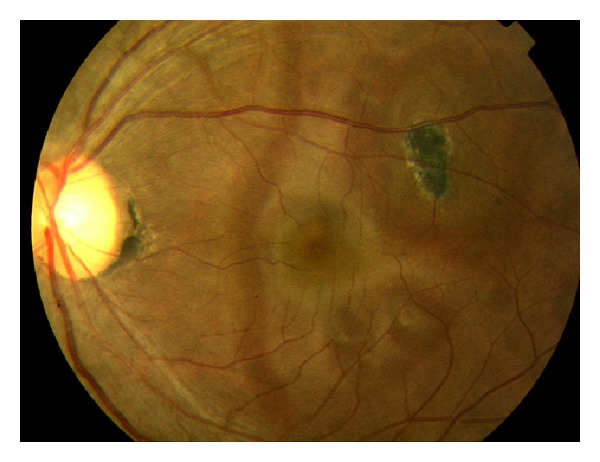
Photo courtesy of Bruce Rivers, M.D. and Madigan Army Medical Center.

On examination, patients present with anterior segment cell, vitritis, and acute choroidal lesions of the macula. Cystoid macular edema and choroidal neovascularization may result from these lesions, both contributing to vision loss. Retinal pigment epithelium metaplasia and fibrous scarring are additional causes of vision loss. The classic lesions of multifocal choroiditis are 50–100 micron punched out chorioretinal scars with pigmented borders in the posterior pole (Figure 3). These lesions appear similar to ocular histoplasmosis; however, with the present of vitritis, ocular histoplasmosis is excluded [15]. Acute lesions are typically yellow-white and located at the outer retina and choroid. Choroidal neovascularization is a frequent complication in up to 33% of patients [15]. 

Fluorescein angiography may highlight lesions not visible clinically on exam. Acute lesions show early hypofluorescence and late hyperfluorescence. Cystoid macular edema may be associated with these acute lesions, while choroidal neovascularization may be associated with the juxtapapillary scars and deep macular scars [14]. Spectral-domain optical coherence tomography may be used to distinguish CNV from MFC versus CNV from pathologic myopia. In MFC, SD-OCT shows drusen-like material between the RPE and Bruch's membrane, vitreous cells, and localized choroidal hyperreflectivity. In contrast, pathologic myopia-related CNV shows none of these findings on SD-OCT [16]. Humphrey visual field often demonstrates an enlarged blind spot and in some cases peripheral visual field loss that does not correspond to areas of acute choroiditis [13]. Electrophysiological testing shows variable results with some patients showing reduction in a and b wave amplitudes and other patients showing normal results. If a multifocal electroretinogram is performed, it may show that the macular region is greater affected than the periphery [13].

Treatment of MCP involves corticosteroids (topical, periocular, and systemic) and steroid-sparing drugs due to its recurrence. Early in the course of the disease, systemic or periocular steroids are effective in controlling the disease. Late disease stages that include choroidal neovascularization and subretinal fibrosis require immunosuppressive agents for better control of the disease. Laser photocoagulation, photodynamic therapy, and anti-VEGF treatment are useful for choroidal neovascularization [14]. Parodi et al. conducted a study of 14 patients, comparing bevacizumab versus photodynamic therapy for CNV in MCP. The results of their study showed greater beneficial effects of visual acuity and central macular thickness in the bevacizumab group [17]. 

## 6. Acute Zonal Occult Outer Retinopathy (AZOOR)

Acute Zonal Occult Outer Retinopathy (AZOOR) is a unilateral or bilateral condition that affects predominantly young myopic females. Early in the disease course, patients present with photopsias and a visual field defect. The visual phenomena described by patients affected with AZOOR is very specific photopsias and movement of colors in the area of visual field loss [18]. The photopsias are often long standing, even after inflammation has subsided. Other conditions associated with AZOOR that Gass categorized as the “AZOOR complex of disorders” include idiopathic blind-spot enlargement syndrome, MEWDS, acute macular neuroretinitis, and multifocal choroiditis [19]. 

On initial examination, patients present with mild vitritis and minimal to no funduscopic changes. An afferent pupillary defect is present in a minority of patients. Visual field loss is often limited to the temporal visual field to include the blind spot. Over time this visual field defect may enlarge and migrate centrally or peripherally. Those patients with large scotomas develop retinal pigment epithelium atrophy and pigment clumping, also in the area corresponding to the photopsias [19]. Fluorescein angiography is normal when there are no fundus changes or RPE abnormalities. Electrophysiology displays a consistent pattern of inner retinal dysfunction and RPE dysfunction. Electroretinogram shows a delayed 30 Hz flicker and a reduction in the EOG light rise. Later in the disease course, atrophy of the photoreceptors, narrowing of arterioles, and pigment migration in a bone spicule pattern may occur in those patients with progressive disease [19]. 

Pathologically, the primary lesion in AZOOR is a photoreceptor outer segment dysfunction [20]. Approximately, one-third of patients with AZOOR develop recurrence. In these patients, the leading edge of reactivation displays a gray intraretinal ring; this variant of AZOOR is also called acute annular outer retinopathy. The cause of AZOOR remains unknown. Gass has shown a 28% incidence of autoimmune disease to include Hashimoto's thyroiditis and relapsing transverse myelopathy; however, infectious or viral etiologies cannot be excluded [20]. 

No treatment has shown to effectively treat AZOOR. While one-third of patients may develop recurrence and portend a poorer visual outcome, the majority of patients have one episode with good visual recovery. According to Gass' series of patients 88% retain vision of 20/40 or better once the disease has stabilized. In Gass' same patient population, improvement of the patient's vision occurred 6 months from initial presentation [13].

## 7. Birdshot Retinochoroidopathy (Vitiliginous Chorioretinitis)

Birdshot chorioretinitis is a bilateral condition that affects women more than men, in the fourth to sixth decade of life. Patients often present with nyctalopia, floaters, photopsias, and decreased vision [21]. Many patients will complain of poor vision out of proportion to the visual acuity loss. Because the early stages may have faint to mild inflammation, the patient's complaint may be dismissed, leading to a delay in the diagnosis. Typically, a patient's insistence on decreased night vision, paracentral scotomas, and diminished color vision prompts a more thorough evaluation and eventual diagnosis. Alternatively, while some patients detect the disease processes earlier than their doctor, other patients have no complaints until the disease is advanced to include prominent retinal-choroidal lesions, vitritis, and cystoid macular edema [21].

**Figure 4:**
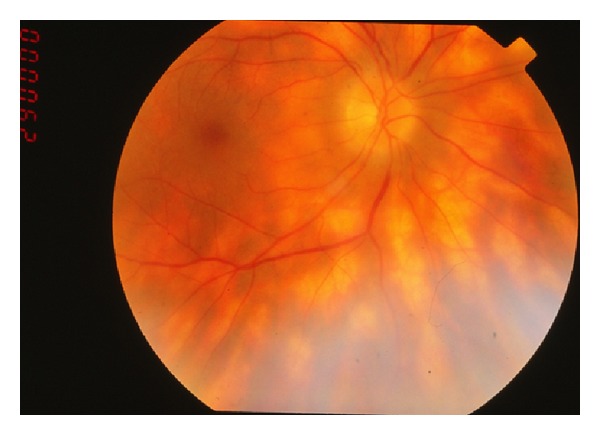
Photo courtesy of Gary Holland, M.D.

On examination, vitritis is uniformly present. Conversely, anterior segment inflammation is generally absent, and posterior synechia does not occur. Prominent vitreous haze or focal vitreous opacities are an exception to the norm. The most prevalent and characteristic findings are yellowish lesions at the level of the deep retina, that radiate out from the optic nerve in a shotgun fashion [22] (see Figure 4). Generally, the spots are more prominent of the nasal retina and evenly are distributed bilaterally. Retinal vasculitis is typical of the disease process; however, it manifests as arteriolar narrowing as opposed to retinal vascular hemorrhage or exudation. Late and chronic disease signs include macular edema and optic nerve pallor [22].

**Figure 5:**
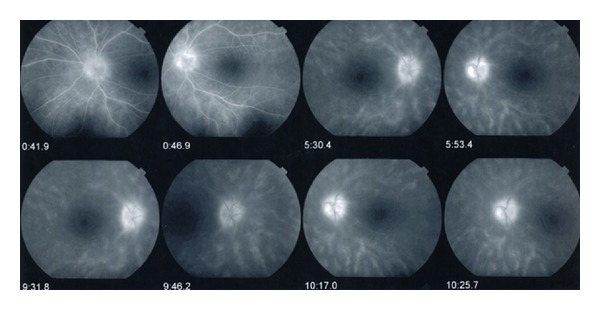
Photo courtesy of Gary Holland, M.D.

Fluorescein angiography usually does not display the birdshot lesions but will highlight the cystoid macular edema, optic nerve head leakage, and retinal vasculitis that may be present [23]. In addition circulation times are often delayed (see Figure 5) and the vessels will empty dye much quicker than a normal eye. This fluorescein angiographic phenomenon, called “quenching,” is a unique feature in Birdshot retinochoroidopathy. As opposed to fluorescein angiogram, ICG angiography displays well the birdshot lesions as areas of blockage in the early to midphase of the angiogram [23]. The lesions radiate along the large choroidal veins. Fundus autofluorescence may be used to demonstrate the RPE atrophy, which is hard to be seen by other means of investigation [24]. Significantly, RPE atrophy in the macula may be an important cause of poor central visual acuity in eyes with birdshot chorioretinopathy [24]. Electroretinogram shows moderate-to-severe depression of rod and cone functions. The key parameter is the 30 Hz flicker implicit time, which is abnormal in 70% of patients at baseline [25]. According to Comander et al., a normal implicit time is correlated with the chance that a patient can be successfully tapered from systemic therapy without recurrence [25]. Visual field testing reveals an overall global depression and often paracentral scotoma [23]. 

Almost 100% of patients with Birdshot are HLA-A29 positive [21]. Because this correlation is so high, a positive HLA confirmation is often considered necessary for confirmation of the diagnosis. Of patients with HLA-A29 associated birdshot, Kuiper et al. showed that when 23 immune mediators in paired aqueous humor and serum samples were tested, IL-17 was consistently elevated in the aqueous humor [26]. This may suggest that birdshot chorioretinopathy is an autoimmune inflammatory disease restricted to the eye and associated with elevated IL-17 [26]. 

 Loss of retinal function is diffuse in birdshot chorioretinopathy as opposed to focal as in many white dot syndromes [23]. The etiology of this global retinal dysfunction may be both secondary to chronic hypoperfusion and changes in the retina pigment epithelium and choroid. The majority of patients have a recurrent course marked by multiple exacerbations and remissions. Vision loss is attributed to cystoid macular edema (one-third of patients), optic atrophy, and rarely choroidal neovascularization. The majority of vision loss is associated with vessel attenuation and optic nerve atrophy [23].

Systemic treatment modalities include steroid-sparing drugs, cyclosporine, mycophenolate, methotrexate, and IVIG [27]. Artornsombudh et al. report treating 22 refractory birdshot chorioretinopathy patients with infliximab over a 7-year period. 88.9% of these patients achieved control of inflammation at the 1-year followup and maintained control throughout the study [28]. Low dose methotrexate has also been shown to be more effective in improving visual acuity in birdshot patients compared to untreated patients and corticosteroid-based treatment regimens [29]. Local treatment modalities include periocular corticosteroid injections for cystoid macular edema and intraocular steroid implants for control of inflammation [22]. Intravitreal sustained-release fluocinolone acetonide device has been used with success by Rothova et al. in a 22-patient study who were all HLA-A29+ [29]. Results of the fluocinolone implant proved effective in improving vision and controlling inflammation without the use of systemic therapy; however, 100% of the patients developed ocular hypertension that required either pressure-lowering therapy or glaucoma surgery by 12 months [29]. 

## 8. Serpiginous Choroidopathy

Serpiginous choroidopathy is an asymmetric bilateral condition that affects healthy patients in the second to sixth decade of life. It is marked by chronic and progressive inflammation of the inner half of the choroid and RPE. Recurrence is very common in serpiginous and may occur weeks to years after the initial event. Presenting symptoms are marked by blurry vision, photopsias, paracentral scotomas, metamorphopsia, and visual field loss [30].

**Figure 6:**
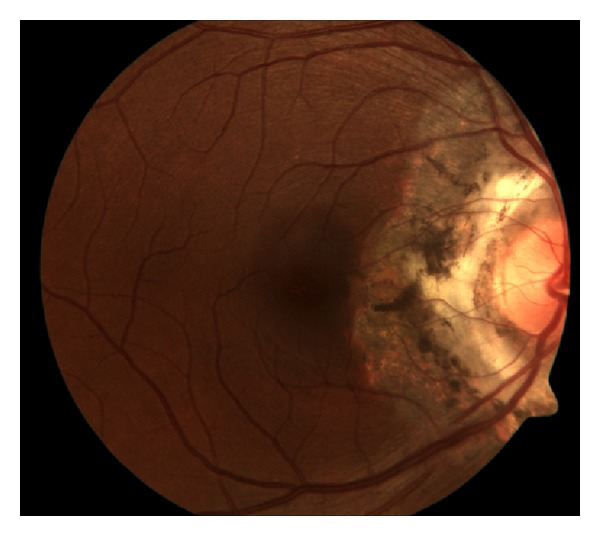
Photo courtesy of Gary Holland, M.D.

On examination, anterior segment inflammation is absent, and vitritis, if present, is usually mild. The pattern of chorioretinal scaring represents a serpiginous or geographic pattern limited to the macula and peripapillary region (see Figure 6). These jigsaw puzzle shaped lesions are deep, located at the level of the choriocapillaris and retinal pigment epithelium [30]. Active lesions may be associated with subretinal fluid and are typically located adjacent to old atrophic lesions [31]. These atrophic lesions involve the retina, retinal pigment epithelium, and the choriocapillaris, often causing subretinal fibrosis [31]. 

**Figure 7:**
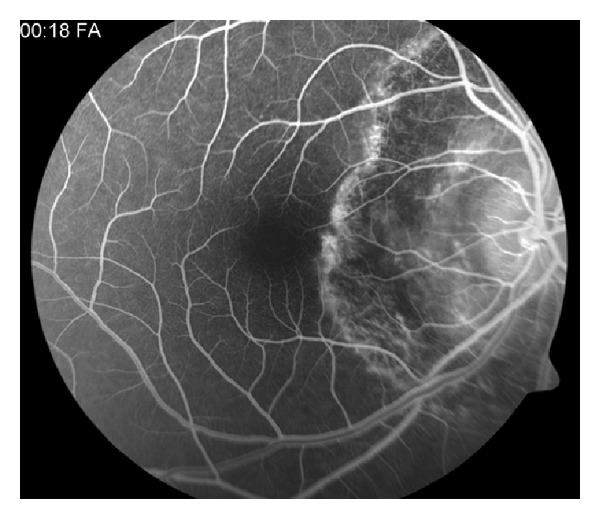
Photo courtesy of Gary Holland, M.D.

Fluorescein angiography shows early hypofluorescence and late hyperfluorescence at the boarders of active lesions [32] (Figure 7). ICG angiography involves 4 separate stages: (1) hypofluorescent lesions in the subclinical or choroidal stage, (2) hypofluorescent lesions in the active stage, (3) hyperfluorescence in the healing and subhealing stage, and (4) hypoflouorescent lesions with early defined margins in the inactive stage [32]. ERG and EOG studies are typically normal [30].

Serpiginous choroidopathy has been associated with an increased incidence of HLA-B7 and levels of retinal S antigen. Herpes virus and factor VIII (Von Willebrand) antigen have also been implicated, yet a definitive etiology remains unknown [14]. Choroidal neovascularization is present in 25% of patients [31]. Systemic immunomodulation to include cyclosporine, cyclophosphamide, and chlorambucil is the preferred first-line treatment. Corticosteroids alone have shown to be not as effective as immunosuppressive agents. Choroidal neovascularization can be treated with laser photocoagulation, photodynamic therapy, or intravitreal anti-VEGF agents [31]. Balaskas et al. showed improvement of CNV associated with serpiginous choroiditis, and Rouvas et al. showed regression of CNV associated with serpiginous choroiditis with no recurrence for over one year [32, 33]. 

The precise etiology of each of these white dot diseases of the retina remains unknown. Some of the white dot syndromes are precipitated by viral infections and immunizations, like APMPPE and MEWDS. Some white dot syndromes are noted in higher incidence with autoimmune diseases, like the AZOOR complex disorders [20]. Other white dot syndromes have strong HLA associations, like birdshot chorioretinopathy and serpiginous choroiditis. And finally, many of the white dot syndromes have similar demographic profiles to include young healthy females with myopia.

Jampol and Becker propose a common genetic hypothesis of autoimmune inflammatory diseases for the eye, whereby the human genome has nondisease specific loci that contribute to the autoimmune disease [34]. Based on Jampol's assertion, certain illnesses, viruses, or environmental factors may cause a patient to develop a white dot syndrome. Additionally, because similar genetic profiles are shared in families, these diseases may show familial clustering [34]. Overall, the various inflammatory chorioretinopathies have several unique features; a small number of them may have some overlapping features such as clustering in women or families with autoimmune diseases. A comprehensive evaluation of these patients should enable arriving at the correct diagnosis and leading to appropriate management.

## Figures and Tables

**Table 1:** 

	ARPE	MEWDS	APMPPE	PIC	MCP	AZOOR	Birdshot	SFU	Serpiginous Choroidopathy
Sex	M = F	F > M	M = F	F > M	F > M	F > M	F > M	F > M	M > F
Age	Young (10 s–30 s)	(20 s–50 s)	(20 s–30 s)	Young (≤40)	(20 s–60 s)	Young women	(40 s–60 s)	(10 s–30 s)	(30 s–60 s)
Laterality	Variable (Uni-75%)	Mostly unilateral	Bilateral	Bilateral	Bilateral	Bilateral but can be asymmetric	Bilateral	Bilateral (asymmteric)	Bilateral but usually asymmetric
Onset	Sudden	Acute	Acute	Sudden	insidious	Sudden	Insidious	Acute	Variable
Viral Prodrome	Variable	Variable	Variable	None	Variable	Variable	None	None	None
Symptoms	Decreased visual acuity or central metamorphopsia or scotoma	Blurred vision, scotomas, photopsias	Blurred vision, scotomata, Photopsia	Decreased central visual acuity, Photopsias, Scotomata	Blurred vision, scotomata, photopsias, and floaters	Visual field defect, blurred vision, photopsias, whitening of vision	Blurred vision, floaters, difficulty with night vision/or color vision, photopsias	Decreased vision	Blurred vision, paracentral or central scotomata, photopsias
Duration	Weeks-Months	Weeks-months	Weeks-Months		Chronic	Weeks to months	Chronic	Chronic	Chronic
Recurrence	Variable	Rare	Rare	Rare	Recurrent	Variable	Recurrent	Recurrent	Recurrent acute lesions lasting weeks-months
Findings	Small hyperpigmented lesions (100–200 *μ*m)	Myopia, small white dots in outer retina/RPE, may coalesce to form patches, disc edema, white/orange granularity at level of fovea and enlargement of blind spot	Multifocal, Flat, Gray-white placoid lesions at the level of posterior pole RPE improving within 1-2 weeks, may have disc swelling	Small (100–300 mm in diameter) multiple gray or yellow, opaque round lesions at the level of the RPE-choroid, scattered throughout the posterior pole, evolve usually into atrophic chorioretinal scars, may be complicated by CNV or subretinal fibrosis	Myopia, Iritis in 50%, Yellow-white lesions replaced by punched out scars, +/− disc swelling	Normal appearing fundus or some RPE mottling or zones with retinitis pigmentosa (RP) appearance	multiple, ill-defined cream-colored lesions at level of outer retina/RPE, patches of depigmentation, otic atrophy and some disc swelling	Blurred and decreased vision	Pseudopodial/geographic zone of gray-yellow discoloration of RPE in peripapillary/macula area with centrifugal extension with active and peripheral edge at the RPE and choriocpillaris
Vit Cells	Mild/none	Mild	Mild	None	Moderate	Normal to mild vitritis	Moderate	AC and Vitreous inflammation	Mild versus absent
FA	Early hyperfluorescence, late staining	Early hyperfluorescence with punctate leisons in wreathlike configuration, late staining with	Early block with late staining in acute phase of disease and window defects in later stage of disease process	Early block with late staining in early phase of disease and window defects in later stage of disease process	Early block with late staining in acute phase of disease and window defects in later stage of disease process	Normal angiography to variable changes to include hyperfluorescence, window defects and optic nerve head leakage.	May have vascular leakage	Yellow-white lesions (50–500 *μ*m) in the posterior pole to midperiphery, RPE hypertrophy, and atrophy and stellate zones of subretinal fibrosis	Hypofluorescent early, borders stain late
ERG/EOG	Normal ERG with Abnormal EOG	Abnormal ERG	Abnormal EOG	Normal-mild changes in ERG	Normal-abnormal ERG	Abnormal ERG	Abnormal rod and cone ERG	Abnormal ERG/EOG	Normal
CME/CNV	None	Rare	None	1/3 develop CNV	CME may be seen with CNV	CME Rare	CME, rare CNV	CME	CNV rare but can occur at margin of chorioretinal atrophy.
Treatment	None	Observation	Observation; consider corticosteroids with CNS involvement	Observation versus oral/periocular corticosteroids	Corticosteroids; photocoagulation. PDT/Anti-VEGF for CNV	No treatment known to improve symptoms	Cyclosporine, Mycophenolate, methotrexate, IVIG	Corticosteroids for CME, immunomodulat-ory therapy	Immunosupression, antivirals, Photocoagulation for CNV
Prognosis	Excellent	Very Good	Good	Good	Generally poor	Good, stabilization of visual fields defects usually within 6 months of onset	Guarded	Guarded	Guarded
Etiology	Unknown	viral	viral	Autoimmune	Viral	Autoimmune	Autoimmune	Autoimmune	viral, autoimmune,
Sequelae		Mild RPE changes	RPE mottling/ depigmentation	Scarring, CNV (30%)	Punched out scars,		CME, rare CNV	Venous sheathing, disc edema	RPE mottling, scarring, loss of choriocapillaris, CNV
HLA	None	None	HLA-B7, HLA-DR2	None	None	None	HLA-A29 (strong)		HLA-B7, S-antigen
